# Effects of a Fence on Pollutant Dispersion in a Boundary Layer Exposed to a Rural-to-Urban Transition

**DOI:** 10.1007/s10546-018-0367-1

**Published:** 2018-07-03

**Authors:** H. E. Eisma, J. M. Tomas, M. J. B. M. Pourquie, G. E. Elsinga, H. J. J. Jonker, J. Westerweel

**Affiliations:** 1 Laboratory for Aero and Hydrodynamics, Department Process and Energy, Delft University, Leeghwaterstraat 21, 2628 CA Delft, The Netherlands; 2 Department Geoscience and Remote Sensing, Delft University, PO Box 5048, 2600 GA Delft, The Netherlands

**Keywords:** Laser-induced fluorescence, Large-eddy simulation, Mixing-length model, Pollutant dispersion, Stereoscopic particle-image velocimetry

## Abstract

Simultaneous particle-image velocimetry and laser-induced fluorescence combined with large-eddy simulations are used to investigate the flow and pollutant dispersion behaviour in a rural-to-urban roughness transition. The urban roughness is characterized by an array of cubical obstacles in an aligned arrangement. A plane fence is added one obstacle height *h* upstream of the urban roughness elements, with three different fence heights considered. A smooth-wall turbulent boundary layer with a depth of 10*h* is used as the approaching flow, and a passive tracer is released from a uniform line source 1*h* upstream of the fence. A shear layer is formed at the top of the fence, which increases in strength for the higher fence cases, resulting in a deeper internal boundary layer (IBL). It is found that the mean flow for the rural-to-urban transition can be described by means of a mixing-length model provided that the transitional effects are accounted for. The mixing-length formulation for sparse urban canopies, as found in the literature, is extended to take into account the blockage effect in dense canopies. Additionally, the average mean concentration field is found to scale with the IBL depth and the bulk velocity in the IBL.

## Introduction

As a result of the worldwide increase in urbanization, more pollutant sources, e.g. from power generation, households, and traffic, are present near densely populated urban areas. Urban air pollution is regarded as carcinogenic to human health by the World Health Organization (2013). Dispersion models can help in taking effective measures to reduce the effects of air pollution, and the development of such models requires proper understanding of dispersion processes in the urban environment. Previous research has focused primarily on dispersion phenomena in so-called fully-developed conditions over areas with uniform properties (Cheng and Castro 2002). However, in real life, the urban roughness often varies in space, which forces the overlying flow to adapt continuously to the new surface conditions. Currently, only a few experimental and numerical studies deal with explicitly resolved roughness transitions (Lee et al. 2011; Cheng and Porté-Agel 2015), while fewer studies consider the effects of a roughness transition on pollutant dispersion behaviour. Recently, Tomas et al. (2017) presented results of a combined experimental and numerical approach on the flow and concentration statistics for an urban boundary layer experiencing a rural-to-urban roughness transition for various spanwise aspect ratios of the roughness elements.

An urban canopy model is described by Coceal and Belcher (2004), for which the flow around urban canopies is modelled solely based upon the geometry of the local roughness elements. This model closely follows that proposed by Belcher et al. (2003), who considered the parametrization of the adjustment region of a turbulent boundary layer to a change in surface roughness. The value of these models is that only geometrical information is required in order to characterize the flow in urban canopies. However, as noted by Coceal and Belcher (2004), the models usually only give satisfactory results for relatively sparse canopies, i.e. $\lambda _\mathrm{f}<0.25$, where the frontal area index $\lambda _\mathrm{f}=A_\mathrm{f}/A_\mathrm{t}$ is a measure of the roughness density (Wooding et al. 1973), Here, $A_\mathrm{f}$ is the total frontal area of the obstacles, and $A_\mathrm{t}$ is the total top view area.

The current study presents the results of the influence of a sound barrier, modelled as a simple two-dimensional (2D) plane fence, on the flow and pollutant characteristics in urban areas. This case is particularly relevant for frequently encountered situations in daily life, where a sound barrier is located between a highway and urban areas. It is well known that sound levels are effectively reduced by placing such a barrier between highways and urban areas, although the effects of a sound barrier on air quality are largely unknown. Besides this, in most practical situations, it is feasible to adapt to the height of the sound barrier while, for lack of space, there is little choice in locating it at any other position in between the roadway and urban areas. Hence, herein we only vary the fence height.

The flow over a 2D fence has been considered in a number of previous studies, e.g. Orellano and Wengle (2000) and di Mare and Jones (2003), although these did not include the dispersion of a passive scalar. Vinçont et al. (2000) measured the passive scalar fluxes in the wake of a 2D obstacle, where the line source is located at the wall downstream of the obstacle. The highest concentrations were recorded in the wake, which is very different from the case of a sound barrier located downstream of the source (i.e., between the highway emissions and the downstream urban area) as illustrated by the present results. More recent investigations have focused on the air pollution behind a sound barrier (Baldauf et al. 2008; Brechler and Fuka 2014; Pournazeri and Princevac 2015), although there is a consensus that the problem needs further investigation.

Our study is an extension of Tomas et al. (2017), who considered the flow and pollutant dispersion over a rural-to-urban roughness transition. They considered roughness elements with different lateral aspect ratios, from cubic obstacles to elongated obstacles, including two-dimensional (2D) ribs. Just upstream of the obstacles a line source was located from which a pollutant was introduced into the flow. The present set-up is similar; the urban canopy consists of cubical obstacles in an aligned arrangement. However, here the aspect ratio of the urban roughness elements $= 1$, while a fence is added in between the line source and the urban areas. In addition, three different fence heights are investigated, while we limit ourselves to the case where the fence and the array of cubes are normal to the wind direction. This configuration is considered most relevant as it represents the case where the pollutant flux has the largest effect on the urban area (e.g., for a ring-road around a city).^1^ Also, this configuration is easiest to implement in a water tunnel with sidewalls that run parallel to the main flow direction, and which impose the boundary conditions of the flow in the lateral direction.

To assess the influence of a sound barrier on the flow and dispersion mechanisms, simultaneous stereoscopic particle-image velocimetry (PIV) and laser-induced fluorescence (LIF) measurements were performed. These experimental results are combined and compared with the results from large-eddy simulation (LES). The objective is to quantify the effect of a fence on the flow field and concentration field for a rural-to-urban roughness transition in terms of velocity statistics, internal boundary-layer (IBL) depth, and pollutant dispersion. For practical purposes the experimental data are limited to a symmetry plane for the mean flow (see Sect. 2), while the LES provides complementary information on the full three-dimensional (3D) structure of the flow, in addition to a much larger range of obstacle rows in the downstream direction. The experimental data from this specific measurement plane are considered to be sufficient to validate the LES results, in addition to comparison with the no-fence case of Tomas et al. (2017). In Tomas et al. (2016, 2017) the LES was used to investigate the 3D character of the canopy flow for obstacles with lateral aspect ratios ranging from 1:1 (cubical obstacles) to 1:8, and for lateral bars (i.e., a pure 2D case). While the canopy flow is 3D, the IBL almost directly above the canopy is essentially homogeneous in the lateral direction, and shows the same qualitative downstream development as for the purely 2D case (with the exception of the 1:2 aspect ratio obstacles). Hence, the measurement and comparison of the present observations in a single plane (i.e., $y = 0$) is considered to be adequate for the present analysis of the characteristics of the IBL for different fence heights and cubical obstacles.

Furthermore, the essential variables are identified and combined into a low-order model for flow and pollutant dispersion for dense urban canopies, similar to the sparse urban canopy model of Coceal and Belcher (2004). Finally, the influence of the IBL, which forms above the urban roughness elements, on the scaling of the mean concentration field is discussed.

The paper is organized as follows: in Sect. 2a short description of the considered cases, experimental set-up, measurement techniques, and numerical method is given. Further details on the experimental and numerical set-up are given in Tomas et al. (2017). Section 3 gives a detailed comparison of the approaching flow conditions, the mean flow statistics, and the turbulent flow field for different fence heights, while Sect. 4 presents the results on pollutant dispersion. In Sect. 5 an updated mixing-length model is proposed that takes into account the development of the IBL, and a conceptual picture is presented on the scaling behaviour and the parametrization of the mean concentration fields. Finally, in Sect. 6 the conclusions are presented.

## Methods

### Considered Cases

**Fig. 1:**
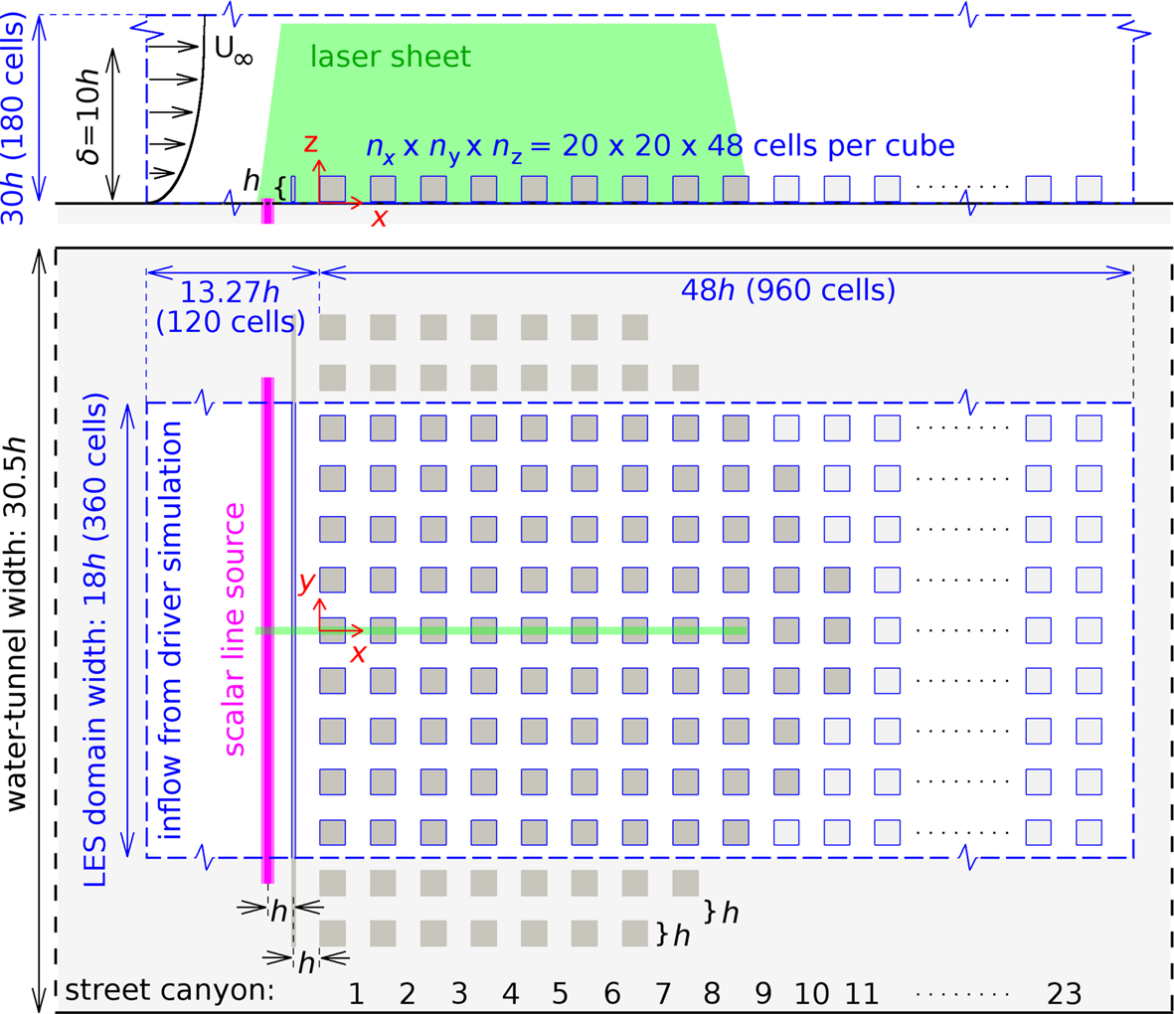
A schematic overview of the experimental and numerical set-up; side view (top) and top view (bottom). The experimental set-up is shown by black lines and dark grey obstacles, while the numerical domain is shown by blue lines and light grey obstacles. A fence is shown as a thin line between the pink line source and the obstacles. The green line represents the laser sheet The figure is adapted from Tomas et al. (2017)

In the experiment and simulation we use the same upstream flow conditions and configuration as in Tomas et al. (2017): a smooth-wall turbulent boundary layer approaches an urban roughness geometry consisting of an array of cubical obstacles with a size of $h\times h\times h$, and placed in an aligned arrangement. This is representative of common situations found in the Netherlands, where flat farmland exists upstream of a roadway with an urban area downstream of the roadway. The Reynolds number of the approaching flow $Re_{\tau }=u_{\tau }\delta _{99}/\nu$, based on the friction velocity $u_{\tau }$ and the boundary-layer depth $\delta _{99}$, was $2\times 10^3$ in the simulations. In the experiments two different Reynolds numbers were considered, i.e. $Re_{\tau }=2\times 10^3$ and $Re_{\tau }=3.5\times 10^3$. A schematic overview of the combined experimental domain (black lines) and numerical domain (blue lines) is given in Fig. 1. The location $x=0$ corresponds to the upstream walls of the first row of obstacles, while the plane $y=0$ lies in the middle of the domain at the symmetry plane of the obstacles. Each ‘street’ consists of an obstacle row and a ‘street canyon’, with the width of all streets equal to *h*. Given this geometric layout, the plan area density $\lambda _\mathrm{p}=A_\mathrm{p}/A_\mathrm{t}$ was equal to the frontal area density $\lambda _\mathrm{f}$, where $A_\mathrm{t}$ is the total area viewed from the top, $A_\mathrm{p}$ is the total area covered with obstacles. $\lambda =\lambda _\mathrm{f}=\lambda _\mathrm{p} = 0.25$, which is in the skimming-flow regime (Oke 1988).

A line source of a passive tracer is placed 2*h* in front of the urban environment to simulate the emission from a highway as is often found near urban regions. In the experiments the tracer is released from a line source at ground level, while in the simulations the line source is located at $z/h=0.2$. A 2D fence is added 1*h* upstream of the first row of obstacles, simulating the presence of a sound barrier next to a highway. The parameter that is varied is the height of the fence. Cases with different fence heights are indicated as C00 for the ‘no fence’ case, C05 for the 0.5*h* fence, C10 (1.0*h*) and C15 (1.5*h*). All cases are represented in the experiments, while only cases C00 and C05 are simulated with LES.

### Experimental Set-Up

**Fig. 2:**
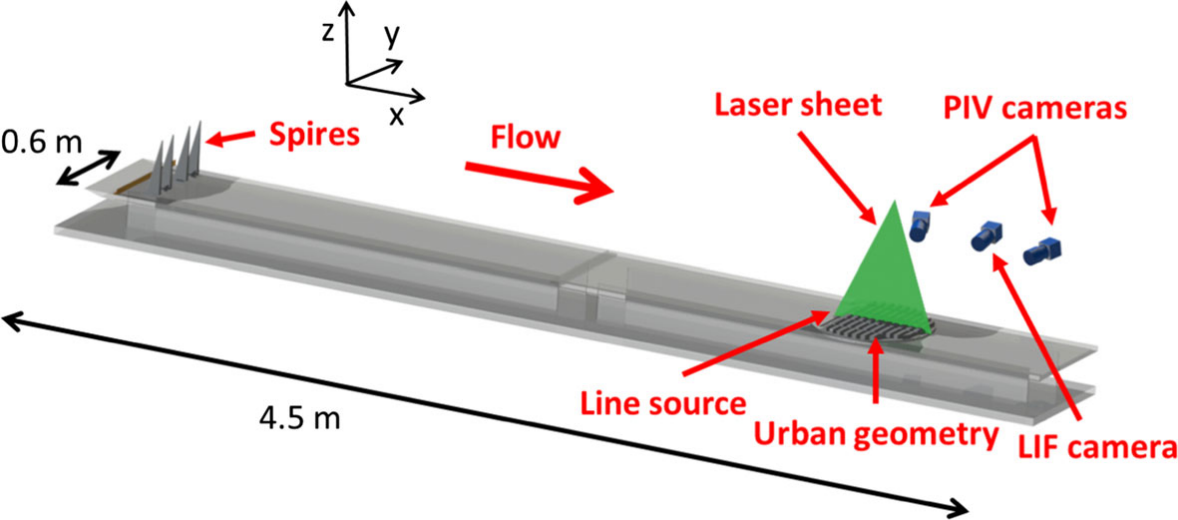
Schematic overview of the experimental set-up indicating the different components The figure is adapted from Tomas et al. (2017)

Here we provide a brief outline of the experimental set-up, summarizing the main characteristics; a more detailed description can be found in Tomas et al. (2017). A schematic overview of the experimental set-up is given in Fig. 2. Experiments were performed in a water-tunnel facility with a cross-sectional size of $0.6\times 0.6\,\mathrm {m^2}$ and a length of 5 m; a false bottom with a length of 4.5 m (see Fig. 2) was mounted 0.17 m above the bottom floor, on which the complete set-up was mounted. An artificially thickened boundary layer over a smooth wall was generated by employing Irwin-type spires (Irwin 1981) and an additional fence positioned upstream of the spires. As in Tomas et al. (2017), a flat terrain was considered as the upstream fetch, and no additional roughness element was added downstream of the spires. The lateral position of the spires was optimized to achieve a uniform profile of the mean velocity ($< 3\%$ variation in magnitude) in the *y*-direction over more than 90% of the tunnel width at the location of the measurements (Eisma 2017, Fig. 4.4).

The urban model, placed approximately 3.9 m downstream of the spires, was constructed from Plexiglas (poly methyl-methacrylate) blocks with a size of $0.02\times 0.02\times 0.02\,\mathrm {m^3}$ and containing eight street canyons along the streamwise direction. The additional fence was an aluminium L-shaped profile with a length of 0.6 m and a thickness of $2\times 10^{-3}$ m. The gaps between the obstacles and the tunnel sidewalls (see Fig. 2) accommodate the boundary layer at the tunnel sidewalls.

Simultaneous stereoscopic PIV and LIF measurements were performed in a common measurement plane above and partly inside the urban canopy. The stereoscopic PIV measurements yield all three components of the instantaneous velocity field inside the measurement plane (i.e., 3C-2D data), while the LIF measurements yield the instantaneous concentration field within the same measurement plane (2D data). For the PIV measurements the flow was seeded with 10-$\upmu$m diameter neutrally-buoyant tracer particles (Sphericell). The $y=0$ plane was illuminated using a double pulsed Nd:YAG laser (Spectra-Physics, Quanta Ray, Santa Clara, California) operating at a wavelength of 532 nm. Each laser pulse has a duration of 5−10 ns (Adrian and Westerweel 2011), with a time delay of $780~\upmu \hbox {s}$ between the two laser pulses, with a resulting field-of-view approximately $0.43\times 0.25\,\mathrm {m^2}$. The thickness of the resulting laser sheet was approximately 1 mm in the measurement area. The digital particle image recordings were made using two high resolution CCD cameras (Image LX 16M, LaVision), equipped with Micro-Nikkor F105 mm objectives and Scheimpflug adapters (Prasad and Jensen 1995). Both objectives operated at an aperture number $f^{\#}=8$. The total separation angle between the PIV cameras was set to $56^{\circ }$, while both cameras had a vertical inclination of $10^{\circ }$ to increase the field-of-view inside the canopy. LIF measurements were made by adding a third camera (Image LX 16M, LaVision) also equipped with a Micro-Nikkor F105 mm objective operating at an aperture number $f^{\#}=2.8$. This camera also had a slight vertical inclination of about $10^{\circ }$ to provide a better view into the canopy. The fluorescent signal (LIF) and the scattered light from the tracer particles (PIV) were separated by employing short-pass optical filters (PIV) and a long-pass optical filter (LIF).

Given that the plane $y = 0$ is a symmetry plane, the mean out-of-plane displacement is also zero (or at least very small) in this plane; hence, this particular measurement plane allows us to use a thin light sheet, and achieve a high spatial resolution for the PIV data, both for the in-plane as well as for the out-of-plane dimensions.

Data acquisition, image calibration, and analysis of the PIV images were performed using the commercial software package Davis 8.3. The PIV images were interrogated using a multi-pass interrogation technique, where the final interrogation windows had a size of $24\times 24$ pixels, corresponding to a spatial resolution of 0.1*h*, with $75\%$ overlap between the neighbouring vectors. Universal outlier detection was performed to replace spurious vectors (Westerweel and Scarano 2005). Each dataset consisted of at least 1000 snapshots acquired at a temporal resolution of 1.44 Hz, and hence reliable and accurate first- and second-order statistics could be obtained as the samples were statistically uncorrelated. The statistical sampling error based on the number of snapshots is less than 1% of the mean freestream velocity magnitude for the estimated mean velocity in the boundary layer and about 10% for the normal stresses and Reynolds stresses, using established uncertainty propagation methods for PIV data (Adrian and Westerweel 2011; Sciacchitano and Wieneke 2016).

LIF measurements were performed by adding Rhodamine WT (Rh-WT, PubChem CID 37718) as the fluorescent dye. In water this dye has a Schmidt number ($Sc=\nu /D$, where $\nu$ is the kinematic viscosity and *D* is the molecular diffusivity of the dye) of about $2.5\times 10^3$. Furthermore, the effective resolution of the concentration fields was 0.004*h* in both directions. The dye was injected 1*h* upstream of the fence by a uniform line source with size $0.01\times 0.4\,\mathrm {m^2}$ ($L_x\times L_y$) at ground level. A syringe pump system was used to inject a concentrated solution of Rhodamine WT at a constant flow rate of $3\, \mathrm {ml\, s^{-1}}$. Calibration of the LIF images was performed by placing a small Plexiglas container with several known uniform concentrations, in the measurement domain. The emission velocity at which the dye is injected is 0.75 mm $\hbox {s}^{-1}$, more than two orders of magnitude smaller than the typical velocity magnitude ($\approx 0.25\,\hbox {ms}^{-1}$) in the recirculating region upstream of the fence. Hence, we expect that the emission rate does not affect the dispersion.

### Numerical Set-Up

The LES set-up is the same as in Tomas et al. (2016, 2017) except that only neutrally-buoyant conditions and cubical roughness elements are considered. In addition, a fence is placed in front of the urban area. Here, the main characteristics of the simulations are given, while further details can be found in Tomas et al. (2016).

#### Governing Equations and Numerical Method

The filtered continuity equation, the filtered Navier–Stokes equations, and the filtered transport equation for scalar concentrations are,

1 $$\begin{aligned} \frac{\partial {\widetilde{u}}_i}{\partial x_i}=&\,0, \end{aligned}$$

2 $$\begin{aligned} \frac{\partial {\widetilde{u}}_i}{\partial t} =&-\frac{\partial }{\partial x_j}\left( {\widetilde{u}}_i{\widetilde{u}}_j\right) -\frac{\partial }{\partial x_i}\left( \frac{{\widetilde{p}}+\tau _{kk}/3}{\rho }\right) + \nu \frac{\partial ^2{\widetilde{u}}_i}{\partial x_j^2} +\frac{\partial }{\partial x_j}\left( 2\nu _{sgs}S_{ij}\right) , \end{aligned}$$

3 $$\begin{aligned} \frac{\partial \widetilde{c^*}}{\partial t} =&-\frac{\partial }{\partial x_j}\left( \widetilde{c^*}{\widetilde{u}}_j\right) + \frac{\nu }{Sc}\frac{\partial ^2\widetilde{c^*}}{\partial x_j^2} +\frac{\partial }{\partial x_j}\left( \frac{\nu _{sgs}}{Sc_{sgs}}\frac{\partial \widetilde{c^*}}{\partial x_j}\right) + \mathcal {S}, \end{aligned}$$

where $\widetilde{(..)}$ denotes filtered quantities, $\left( {\widetilde{p}}+\tau _{kk}/3\right) /\rho$ is the modified pressure, $\tau _{kk}$ is the trace of the subgrid-scale (SGS) stress tensor, $\nu$ is the fluid kinematic viscosity, $\nu _{sgs}$ is the SGS viscosity, $Sc_{sgs}$ is the SGS Schmidt number, $S_{ij}=\frac{1}{2}\left( \frac{\partial {\widetilde{u}}_i}{\partial x_j} + \frac{\partial {\widetilde{u}}_j}{\partial x_i}\right)$ is the rate of strain tensor and $\mathcal {S}$ is a source term. The eddy-viscosity SGS model (Vreman 2004), $\tau _{ij}/\rho = \widetilde{u_iu_j} - {\widetilde{u}}_i{\widetilde{u}}_j=-2\nu _{sgs}S_{ij}$, where $\tau$ is the SGS stress tensor, is already incorporated in Eqs. 2 and 3. Equation 3 describes the transport equation for the pollutant concentration $c^*$. Hereafter the $\widetilde{(..)}$ symbol is omitted for clarity, while the $\overline{(..)}$ symbol represents temporal averaging, and the ${\langle ..\rangle }$ symbol represents spatial averaging.

The equations of motion are solved using second-order central differencing for the spatial derivatives and an explicit third-order Runge-Kutta method for time integration. For the scalar concentration field the second-order $\kappa$ scheme is used to ensure monotonicity (Hundsdorfer et al. 1995). The simulations are wall-resolved, so no use is made of wall functions. The Schmidt number *Sc* is 0.71, and $Sc_{sgs}$ is set to 0.9. The code has been used previously to simulate turbulent flow over a surface-mounted fence, showing good agreement with experimental data (Tomas et al. 2015a, b).

#### Domain and Boundary Conditions

Figure 1 shows the LES domain (in blue) together with the applied number of grid cells in each direction. At the ground and the obstacle walls no-slip conditions were applied, with velocity and concentration fields assumed to be periodic in the spanwise direction. Furthermore, the smooth-wall turbulent boundary layer imposed at the inlet was generated in a separate precursor simulation using the rescaling method proposed by Lund et al. (1998). At the outlet a convective outflow boundary condition was applied for both velocity and concentration. Furthermore, at the top wall free-slip conditions were assumed for the horizontal velocity components. In addition, a small vertical outflow velocity was applied that corresponds to the outflow velocity used in the precursor simulation to achieve a zero pressure-gradient boundary layer; see Tomas et al. (2015b) for details. The computational grid, boundary conditions, as well as the inflow turbulent boundary layer, are the same as for the neutrally-buoyant case described in Tomas et al. (2016). The main characteristics of the numerical grid are summarized in Table 1.

**Table 1:** The main characteristics of the LES numerical grid, with a domain size of $L_x\times L_y\times L_z$ containing $N_x\times N_y \times N_z$ grid points

$L_x$	$L_y$	$L_z$	$N_x$	$N_y$	$N_z$	$\varDelta x_{\mathrm{min}}$	$\varDelta y$	$\varDelta z_{\mathrm{min}}$
61.47*h*	18*h*	30*h*	1080	360	180	0.050*h*	0.050*h*	0.010*h*

For the *x*- and *z*-directions a non-equidistant grid is used with smallest grid dimensions $\varDelta x_{\mathrm{min}}$ and $\varDelta z_{\mathrm{min}}$, respectively; $\varDelta y$ is the equidistant grid dimension in the *y*-direction. Here *h* is the fence height. Further details are given in Tomas et al. (2016)

#### Statistics

The simulations with a turbulent inflow started from a statistically steady solution generated with a steady mean inflow profile. A constant timestep of 0.0156*T* was used, where $T=h/U_h$, and $U_h$ is the relevant velocity scale, as described in Sect. 3.1. The simulations ran for at least 780*T* before statistics were computed, long enough to ensure a steady state. Statistics were computed for a duration of at least 780*T* with a sampling interval of 0.31*T* resulting in converged results. This duration corresponds to approximately 125 uncorrelated samples in the experiment. The simulations were well-resolved, such that the average subgrid stress $\overline{-2\nu _{sgs}S_{13}}$ did not exceed 6% of the total Reynolds stress, as shown by Tomas et al. (2016). Therefore, only the resolved statistics are shown in the subsequent sections.

## Results—Flow Statistics

### Approaching Flow Conditions

**Fig. 3:**
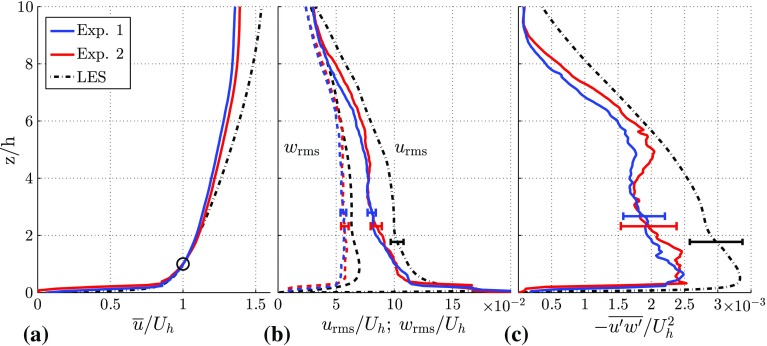
Properties of the approach flow boundary layer; mean streamwise velocity component $\bar{u}$ (**a**), r.m.s. of the velocity components (**b**), and Reynolds stress $-\overline{u'w'}$ (**c**). The circle indicates $U_h\equiv \bar{u}|_{z=h}$. The error bars represent the statistical mean ± the standard error of the mean

Here, a comparison is given of the boundary layer of the approaching flow in the experiments and the simulations. Several studies indicate that the approaching flow conditions influence the flow and dispersion characteristics over obstacles (Castro 1979; Savory et al. 2013; Blackman et al. 2015). All profiles that are given in the subsequent sections are normalized with the undisturbed velocity at obstacle height, $U_h\equiv \bar{u}|_{z=h}$, determined in the measurements of the approach flow. This proved to be the best scaling for the rural-to-urban flows discussed in subsequent sections. However, the velocity at $z=h$ might not be appropriate for scaling the C15 case as the dominant length scale is increased to 1.5*h*. Profiles for the mean streamwise velocity component, the root-mean-square (r.m.s.) velocity fluctuations, and the mean Reynolds stress distribution in the vertical direction are given in Fig. 3. As observed before by Tomas et al. (2017) the agreement between the two different methods is satisfactory, despite the different ways of generating the approaching-flow boundary layer. In the experiments the approaching-flow boundary layer experiences a small favourable pressure gradient due to boundary-layer growth on the walls of the tunnel and the constant cross-sectional area of the tunnel. As a result of the normalization with $U_h$ this is reflected in a lower streamwise velocity component (see Fig. 3a) in the outer region of the boundary layer ($z/h>3$). The difference between the two Reynolds number cases in the experiments is limited, and the streamwise velocity component in the outer layer is reduced marginally for $Re_{\tau }=3.5\times 10^3$. The profiles of the velocity fluctuations $u_{\mathrm {rms}}$ and $w_{\mathrm {rms}}$ depicted in Fig. 3b are similar in shape for both methods. Consistent with previous observations by Tomas et al. (2017), the magnitude of the velocity fluctuations is somewhat reduced as a result of the slightly favourable pressure gradient (Joshi et al. 2014). This effect is slightly larger for the higher Reynolds number. The two methods show similar characteristics for the mean Reynolds stress, as shown in Fig. 3c. Differences are found in the region $2<z/h<5$ where the experimental data shows a reduction in Reynolds stress, which is most likely attributed to the design and arrangement of the spires. Increasing the Reynolds number in the experiments decreases the Reynolds stresses, which is most evident in the region $4<z/h<9$.

The Reynolds number (*Re*) based on $U_{\infty }$ and *h* was around $5\times 10^3$ for the simulations, while the lowest Reynolds number in the experiments was $5.4\times 10^3$. This is in the regime where Reynolds effects are small when the flow over sharp-edged obstacles is considered (Cheng and Castro 2002). The wall-friction Reynolds number $h^+=u_{\tau }h/\nu$ is 194 in the simulations and 213 and 307 in the experiments, which is in the fully-rough regime (Raupach et al. 1991). A summary of the most relevant boundary-layer parameters for different cases in the experiments and the simulations is given in Table 2.

**Table 2:** Summary of the properties of the approaching boundary layer; $\delta ^*$ is the displacement thickness, $\theta$ is the momentum thickness, and *H* is the shape factor

	$\dfrac{\delta _{99}}{h}$	$\dfrac{\theta }{h}$	$\dfrac{\delta ^*}{h}$	*H*	$\dfrac{U_{\infty }}{U_h}$	$\dfrac{u_{\tau }}{U_h}$	$Re_{\tau }$	$h^+$	*Re*
				$\left( \frac{\delta ^*}{\theta }\right)$			$\left( \frac{u_\tau \delta _{99}}{\nu }\right)$	$\left( \frac{u_\tau h}{\nu }\right)$	$\left( \frac{U_{\infty }h}{\nu }\right)$
Exp. 1	11.35	1.10	1.62	1.47	0.72	0.052	$3.5\times 10^3$	307	$8.2\times 10^3$
Exp. 2	9.6	0.95	1.46	1.56	1.41	0.060	$2.0\times 10^3$	213	$5.4\times 10^3$
LES	10.3	1.28	1.78	1.39	1.56	0.059	$2.0\times 10^3$	194	$5.0\times 10^3$

### The Flow Field Over the Urban Canopy

**Fig. 4:**
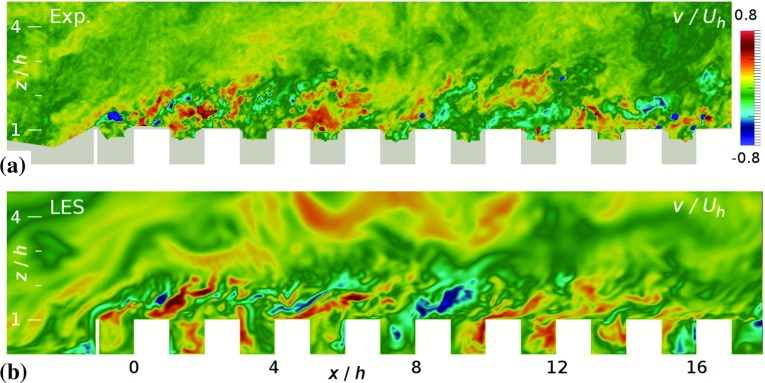
Contours of instantaneous spanwise velocity component $v/U_h$ in the midplane of the C10 case; **a** experiment and **b** simulation. Areas that could not be illuminated or seen in the experiments have been masked in grey and the obstacles are shown in white

Here the rural-to-urban flow fields are compared for the first eight streets in the $y=0$ plane, see Fig. 1, with all velocity statistics normalized with the undisturbed velocity at obstacle height $U_h$. A snapshot of the spanwise velocity component *v* is shown in Fig. 4 for both the experiment and the simulation. Both snapshots clearly indicate the presence of large turbulent structures that are shed from the fence and the roughness elements, with structures growing in size in the downstream direction. Note that the magnitude of the velocity fluctuations in these structures is significantly larger compared to the velocity fluctuations in the approaching flow.

**Fig. 5:**
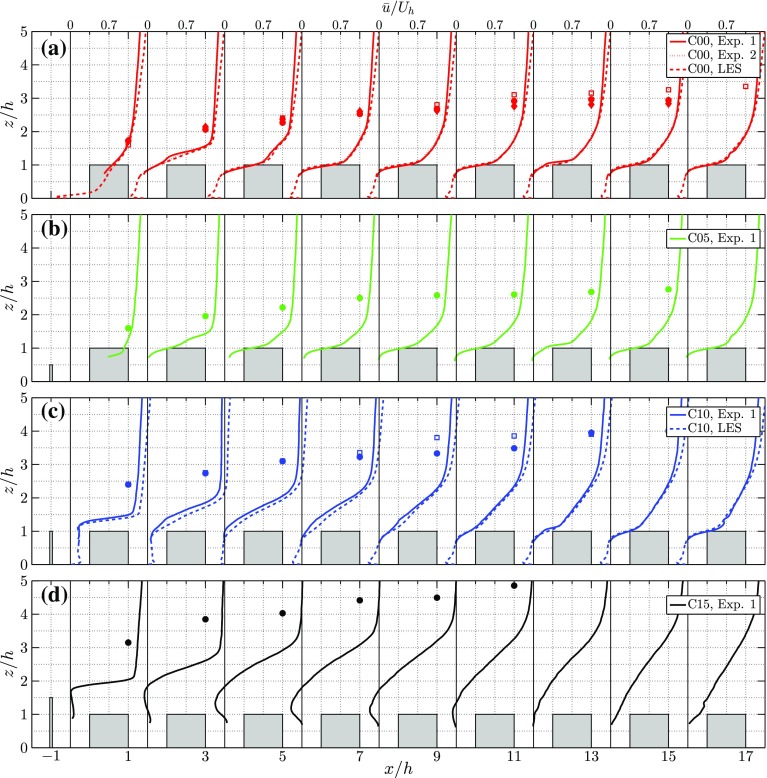
Profiles of the mean streamwise velocity component $\overline{u}/U_h$ in the middle of each street canyon for cases 0*h* (**a**), 0.5*h* (**b**), 1*h* (**c**) and 1.5*h* (**d**), for both the LES and experiments (Exp. 1 in Table 2). Additionally for 0*h* **a** the profiles of $\overline{u}/U_h$ for $h^+=209$ case in the experiments (Exp. 2 in Table 2) is shown as a dotted line. (Please note that the differences between the results of Exp. 1 and 2 in (**a**) are small and that these lines mostly overlap.) The IBL depth, $\delta _i$, is shown by solid markers for the experiments and by open markers for the LES (if available)

In order to make a quantitative comparison between different cases, the mean streamwise profiles for the cases C00, C05, C10 and C15 are shown in Fig. 5. Vertical profiles of the mean streamwise velocity component are shown, starting 0.5*h* behind the fence and subsequently in the middle of each street canyon. Additionally, the IBL depth is shown for the experiments (filled symbols) and the LES (open symbols, if available). The IBL depth $\delta _i$ is found by subtracting the smooth-wall inlet velocity profile from the mean street-averaged velocity field for the roughness transition: ${\Delta } \left<\bar{u}\right>=\left<\bar{u}\right>_{\mathrm {RT}}-\left<\bar{u}\right>_{\mathrm {inlet}}$, noting that $\delta _i$ is defined as the height at which the vertical gradient of ${\Delta } \left<\bar{u}\right>$ reaches zero. A threshold of three times the r.m.s. value of $|\mathrm {d}{\Delta }\left<\bar{u}\right>/\mathrm {d}z|$ outside the canopy region is used to determine this location.

In Fig. 5a the C00 cases are plotted. There is a close agreement between the LES and the experimental results at the lower Reynolds number (C00 Exp. 2), as noted before in Tomas et al. (2017). Additionally, the higher Reynolds number case $h^+=307$ (solid red line) also closely matches the other two curves. The main difference can be found in the outer region of the flow ($z/h>\delta _i$). This small difference can be attributed to the difference in approaching flow conditions, as observed in Fig. 3a. Given the limited capabilities of varying the flow speed of our water tunnel, we could only consider two Reynolds numbers that differ by a factor of two. The close resemblance of the scaled results at these two Reynolds numbers indicates that in this range the Reynolds-number dependence would be rather weak, which appears to be in correspondence with the conclusion of Raupach et al. (1991). The profiles of the streamwise velocity components change noticeably over the first three rows of obstacles while further downstream only minor changes are observed. The differences between cases C05 (Fig. 5b) and C00 are marginal, and the downstream influence of the 0.5*h* fence on the mean streamwise velocity profiles is only limited.

With increasing fence height, the downstream influence of the fence increases. Both the C10 case (Fig. 5c) and the C15 case (Fig. 5d) introduce a very sharp and distinct shear layer just behind the fence, i.e. at $x/h=-0.5h$. Further downstream, this shear layer increases in thickness. From Fig. 5c it is clear that the initial disturbance generated in the C10 case decays and the velocity profile shows similar behaviour as the C00 case in the eighth street. The LES results of the C10 case indicate a slightly higher streamwise velocity above the first few street canyons (Fig. 5c). This can most probably be attributed to the difference in blockage in both methods, as the height of the LES domain (30*h*) is smaller compared to that of the experiments (40*h*). Furthermore, the LES model uses an outflow boundary condition at the top of the domain whereas the water-tunnel facility has a free water surface. Especially the C15 case (Fig. 5d) indicates the presence of a significant recirculation region that extends up to $x/h\approx 12$, inducing backflow at the top of several obstacles. Although this disturbance slowly decays downstream, in the eighth street there is still a significant difference between the C00 and C15 cases. Finally, it is clearly visible that increasing the fence height significantly increases the IBL height.

**Fig. 6:**
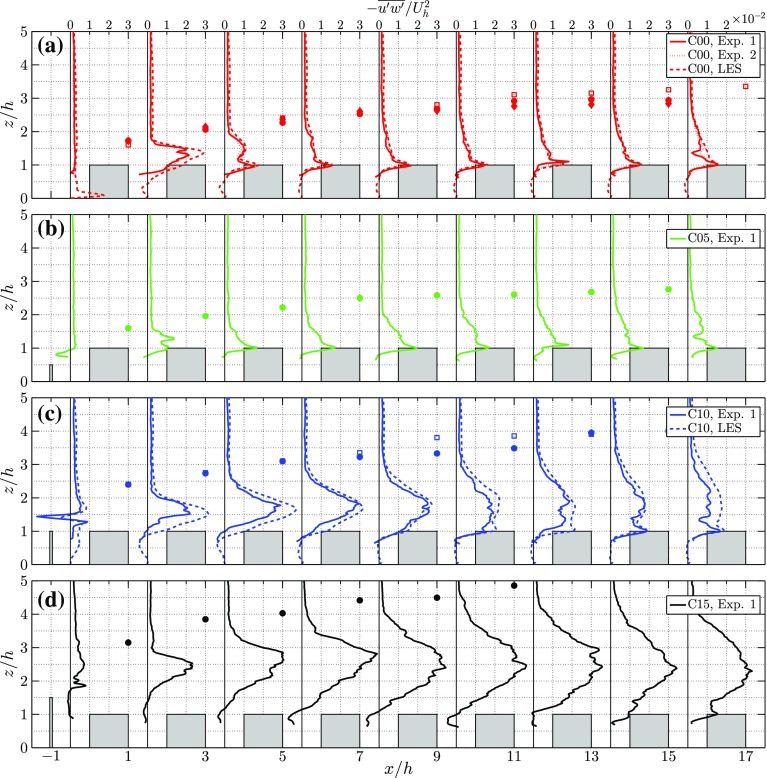
Profiles of the mean Reynolds stress $-\overline{u'w'}/U_h^2$ in the middle of each street canyon for cases 0*h* (**a**), 0.5*h* (**b**), 1*h* (**c**) and 1.5*h* (**d**) for both the LES and experiments (Exp. 1 in Table 2). Additionally for the 0*h* case **a** the profiles of $\overline{u}/U_h$ at $h^+=209$ in the experiments (Exp. 2 in Table 2) is shown as a dotted line. (Please note that the differences between the results of Exp. 1 and 2 in **a** are small and that these lines mostly overlap.) The IBL depth, $\delta _i$, is shown by solid markers for the experiments and by open markers for the LES (if available)

Figure 6 shows the profiles of the mean Reynolds stress $-\overline{u'w'}$ at the corresponing locations, with the overall difference between the experiments and the LES relatively small. Figure 6a shows that both experimental results, i.e. Exp. 1 and Exp. 2, indicate a smaller value of the peak in Reynolds stress than in the LES results, while the location of this peak is similar to the peak location observed in the LES results. This suggests that, as a result of the finite PIV resolution, the Reynolds stress in the experiments is underestimated. An estimate for the fraction of resolved Reynolds stress to the total Reynolds stress is made by considering the ratio between the PIV resolution and the length scale $\Lambda$ of the dominant flow structure (Scarano 2003). As in Tomas et al. (2017), the length scale of the dominant flow structure is calculated from the two-point correlation of *u*. Following this procedure, the resolved part of the Reynolds stress in Fig. 6a is approximately 75 and 83% of the total for Exp. 1 and Exp. 2, respectively. The magnitude of the Reynolds stress in Fig. 6c is smaller in the experiments compared to the LES results, while the shape of the profile is similar. This is a direct consequence of the presence of the fence that introduces a dominant length scale that is even smaller compared to the no fence case, i.e. in the experiments only 55% of the Reynolds stress is resolved in the peak above the first street canyon ($x/h=1.5$). With increasing fence height the peak in $-\overline{u'w'}$ increases, and in the downstream direction this peak becomes wider and decreases in amplitude. For the fence heights up to 1*h*, most of the turbulence in street 8 is produced by the rooftop shear layers, except for the C15 case in which the peak emanating from the fence still dominates.

## Results—Pollutant Dispersion

This section deals with the pollutant dispersion behaviour by investigating concentration fields acquired in both the experiments and the simulations. All concentrations $c^*$ are non-dimensionalized using the reference velocity $U_h$, the obstacle height *h*, the source width $L_y$, the source concentration $c_s$, and the source volume flow rate $\phi _s$ as,

4 $$\begin{aligned} c = \frac{c^*U_h h L_y}{c_s \phi _s}, \end{aligned}$$

noting that $\phi _s / L_y$ is the emission rate per unit source width.

**Fig. 7:**
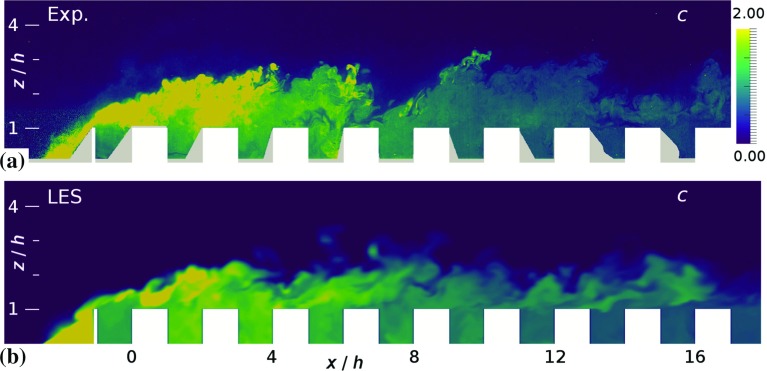
Contours of instantaneous concentration *c* (defined in Eq. 4) in the midplane of case C10; **a** experiment and **b** simulation. The snapshots correspond to the velocity fields shown in Fig. 4. Areas that could not be illuminated or seen in the experiments have been masked in grey and the obstacles are shown in white. The finer scales in the experiment are due to a higher Schmidt number in comparison with that of the simulation

Two snapshots of the concentration fields for both methods for the C10 case are shown in Fig. 7, where both snapshots indicate the strongly intermittent character of the concentration field where a fence is present. As a result, slender plumes of high concentration are visible just above the top of the fence. This is because the line source is positioned close to the fence, i.e. the distance between the fence and the centerline of the source is 1*h* (see Fig. 1). On average a recirculation region is present just in front of the fence (Tomas et al. 2015b). Therefore, the instantaneous occurrence of this recirculating flow structure, largely influenced by the upstream turbulence, is responsible for the intermittent behaviour of the concentration field; pollutants are trapped in the recirculating flow and released over the top of the fence when the recirculation is weak or non-existent. The Schmidt number in the experiments is significantly higher compared to that used in the simulations. However, the turbulent Schmidt number is of the same order of magnitude (Tomas et al. 2017), which is also suggested by a high degree of similarity in the large structures observed in Fig. 7.

### Mean Concentration Fields

**Fig. 8:**
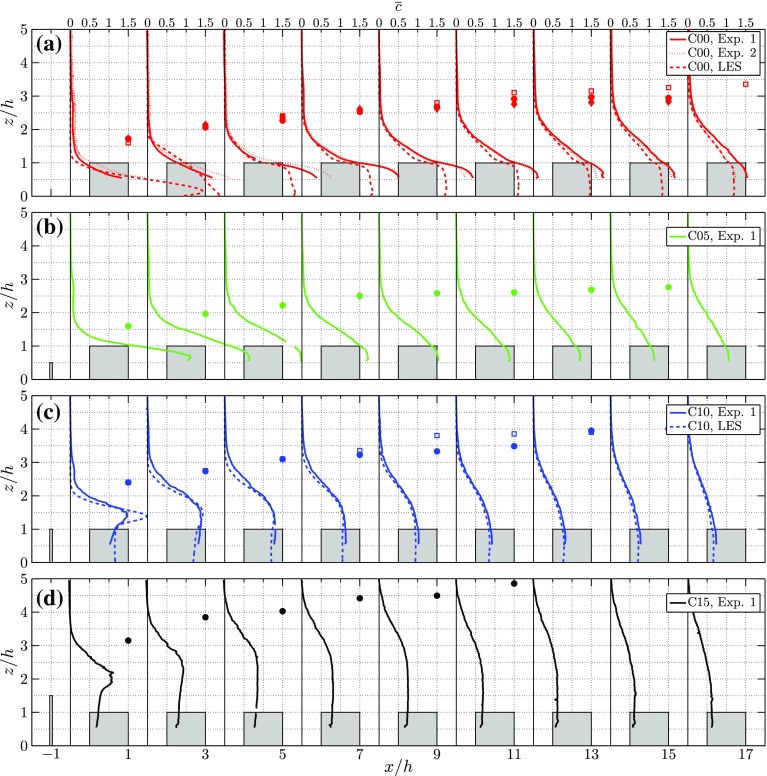
Profiles of the mean concentration $\overline{c}$ in the middle of each street canyon for cases 0*h* (**a**), 0.5*h* (**b**), 1*h* (**c**) and 1.5*h* (**d**). Additionally for the 0*h* case **a** the profiles of $\overline{c}$ at $h^+=209$ in the experiments is shown as the dotted line. The IBL depth, $\delta _i$, is shown by solid markers for the experiments and by open markers for LES (when available)

Figure 8 shows the vertical distribution of the mean concentration starting 0.5*h* upstream of the first row of obstacles and subsequently in the middle of each street canyon for cases C00, C05, C10, and C15. The experimental data are shown by the continuous lines while, when available, the LES data are shown by the dashed lines. The IBL depth $\delta _i$ is represented by the solid markers for the experiment and by the open markers for the LES. Note that in the second street canyon ($x/h=3.5$) no experimental data for Exp. 1 are shown in the region $0.975<z/h<1.13$ due to a dust particle present in the calibration tank at that location, which prevented the LIF calibration at that location.

**Fig. 9:**
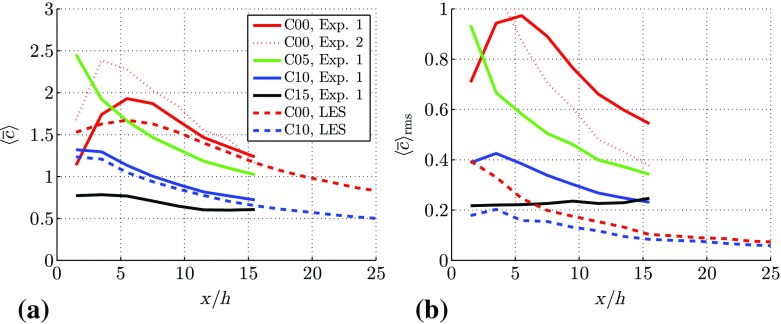
Profiles of **a** the mean canyon concentration $\left<\overline{c}\right>$, and **b** the r.m.s. of the canyon concentration fluctuations $\left<\overline{c}\right>_{\mathrm {rms}}$, in the $y = 0$ plane for different fence heights

From the C00 case, it is clearly seen that the Reynolds number effect in the considered Reynolds number range is small. The profiles of the two experimental datasets at $h^+=209$ and $h^+=307$ are nearly on top of each other in the region $z/h>1$, while minor differences are found inside the canopy region. The most pronounced difference between the experiments and the simulations is found in the first few streets of the canopy (see Fig. 8a), where a significantly higher concentration is found in the experiments. Separate tests on the spanwise homogeneity of the experimental line source reveal a slightly non-uniform flow rate distribution (Tomas et al. 2017). Nevertheless, the average flow rate through the line source is kept constant in time. Despite this difference the shape of the concentration profiles is similar in both methods. Moreover, as a result of the turbulent mixing, this lateral inhomogeneity decreases further downstream, resulting in a match between both methods in the eighth street canyon. The vertical distribution of concentration develops in a similar way to the IBL, and scales with $\delta _i$ (Tomas et al. 2017). For C10 and C15 (see Fig. 8c, d) the concentration profile at $x/h=-0.5$ depicts a peak in concentration just above the fence. In the downstream direction this peak rapidly vanishes and the maximum concentration is found inside the canopy region (i.e. $z/h<1$), similar to the C00 case (Fig. 8a). Due to the strong mixing related to the shear layer emanating from the fence, a region of nearly uniform concentration appears above the street canyon. This behaviour is particularly visible in the C15 case (Fig. 8d). Furthermore, the mean concentration inside the IBL reduces considerably with increasing fence height. Figure 9 shows the streamwise development of the average street-canyon concentration $\left<\overline{c}\right>$ and the r.m.s. of the concentration fluctuations for the plane $y = 0$. Since the street canyon is not completely visible in the experimental results, the average street-canyon concentration is determined only in the upper part of the street canyon.

For the C00 case the mean canyon concentration increases for the first two streets and reaches its maximum in the third-street canyon. This is because a large part of the concentration is advected with high speed through the streamwise streets, thereby passing the first two street canyons. After a few streets the flow velocity is decreased, allowing the pollutants to entrain the recirculation region in the street canyons, which results in an increased canyon concentration. [This is a clear 3D effect that occurs in the ‘no-fence’ case C00 that was studied and reported by Tomas et al. (2017)]. The concentration curve is different once a fence is introduced. Already for the case C05, the streamwise development of the mean canyon concentration is distinctly different from the C00 case, despite the flow fields being quite similar as shown in Figs. 5b and 6b. The maximum canyon concentration is found in the first street canyon for the fence cases, while it monotonically decreases further downstream. The reason is that a fence inhibits the transport of pollutants along streamwise streets. The maximum canyon concentration for cases C05, C10, and C15 is found in the first street canyon; furthermore, with increasing fence height, the maximum of the mean concentration $\left<\overline{c}\right>$ decreases. Finally, the profiles of $\left<c_{\mathrm {rms}}\right>$, depicted in Fig. 9b, indicate a similar streamwise trend as $\left<\overline{c}\right>$. The difference in the magnitude of $\left<c_{\mathrm {rms}}\right>$ between LES and experiments is partly attributed to the higher Schmidt number in the experiments, resulting in higher r.m.s. values for the experimental data since diffusion is less effective in reducing concentration gradients. Additionally, LES uses a so-called $\kappa$ scheme for the advection terms of the transport equation for *c*, which has the tendency to reduce fluctuations (Hundsdorfer et al. 1995).

## Modelling Considerations

Here and in that of a preceding investigation (Tomas et al. 2017) the development of an IBL following a rural-to-urban roughness transition is considered in the presence of a line source upstream of the roughness transition. Such transitions are frequently encountered at the edges of urban areas where a roadway separates a rural and urban area, with or without a sound barrier separating the roadway and urban area. These studies provide detailed information on the development of the IBL and the dispersion of pollutants in the urban area, in particular in the transition region. However, in applications where the dispersion of pollutants is considered over extended areas, the experimental and numerical methods employed here are not feasible; instead one has to rely on appropriate closure models to predict the flow and pollutant dispersion. Although conventional methods perform well over larger scales (e.g., over a neighbourhood or entire city), there is a societal need to have reliable predictions at the scale of individual streets, in particular in the vicinity of roadways, as these may pose considerable health risks to their inhabitants.

### The Mixing-Length Model

Flows over and inside the urban canopies are commonly simulated using models based on the Reynolds-averaged Navier–Stokes equations; however, adequate closure relations are needed to model the Reynolds stresses. One of the most basic closure models of the Reynolds stresses, relating the time-averaged Reynolds shear stress to the gradients of the mean flow, was introduced by Boussinesq (1877) as

5 $$\begin{aligned} -\overline{u'w'}=\nu _T\left( \frac{\partial \overline{u}}{\partial z}+\frac{\partial \overline{w}}{\partial x}\right) , \end{aligned}$$

where the turbulent viscosity $\nu _T$ can be written as (Prandtl 1925)

6 $$\begin{aligned} \nu _T = l_\mathrm{m}^2\left| \frac{\partial \overline{u}}{\partial z} \right| , \end{aligned}$$

where $l_\mathrm{m}$ is an unknown mixing length. This mixing, length model is also commonly used in 1D models of urban canopy flows (Macdonald 2000; Coceal and Belcher 2004). The model of Coceal and Belcher (2004) is basically the non-linear extension of the quasi-linear model that Belcher et al. (2003) proposed to model the adjustment of a rural boundary layer to an urban canopy. Macdonald (2000) proposed the use of a uniform mixing length inside the urban canopy, and a value that increases linearly with height above the canopy, i.e. $l_\mathrm{m}\propto z$. An alternative formulation of the mixing length inside the canopy was proposed by Coceal and Belcher (2004) as,

7 $$\begin{aligned} \frac{1}{l_\mathrm{m}(z)} = \frac{1}{\kappa z}+\frac{1}{l_\mathrm{c}}, \end{aligned}$$

where $\kappa$ is the von Kármán constant, and the spatially-averaged mixing length in the canopy, $\mathrm{c}$, is constant and depends on the thickness of the shear layer emanating from the top of the obstacles, i.e. $h-d$. It is computed as $l_\mathrm{m}(z=h)=\kappa (h-d)$ to ensure a continuous profile. The zero-plane displacement $d=0.72h$ is found by fitting the streamwise velocity profile to the law of the wall,

8 $$\begin{aligned} \frac{\overline{u}}{u_{\tau }} = \frac{1}{\kappa } \mathrm {ln} \left( \frac{z-d}{z_0}\right) , \end{aligned}$$

where $z_0$ is the aerodynamic roughness length. The proposed form of the mixing length by Coceal and Belcher (2004) is motivated by, on the one hand, the sparse canopy case, in which the turbulent eddies scale with the distance from the ground, while on the other hand, the dense canopy limit in which eddies are blocked by the strong shear layer near the roof level of the roughness elements. In the latter case the mixing length above the canopy scales with $l_\mathrm{m}(z)=\kappa (z-d)$, i.e. the displaced mixing-length model (Belcher et al. 2003). Both Macdonald (2000) and Belcher et al. (2003) note that their models are valid for a relatively low packing density (i.e. $\lambda _\mathrm{f}\le 0.25$). Especially at higher packing densities these models are inadequate in predicting the velocity statistics due to the recirculation regions inside the street canyons. Because a similar simple model for dense urban canopies is not available, we consider the possibility of using a mixing-length model for these cases. Cheng and Porté-Agel (2015) present the mixing-length profiles for different $\lambda _\mathrm{f}$ for staggered and aligned arrays of cubical obstacles, for $\lambda _\mathrm{f}\le 0.25$; see also Castro (2017). Here, $\lambda _\mathrm{f} = 0.25$ for the C00 case and 0.5 for the 2D reference case from Tomas et al. (2017).

**Fig. 10:**
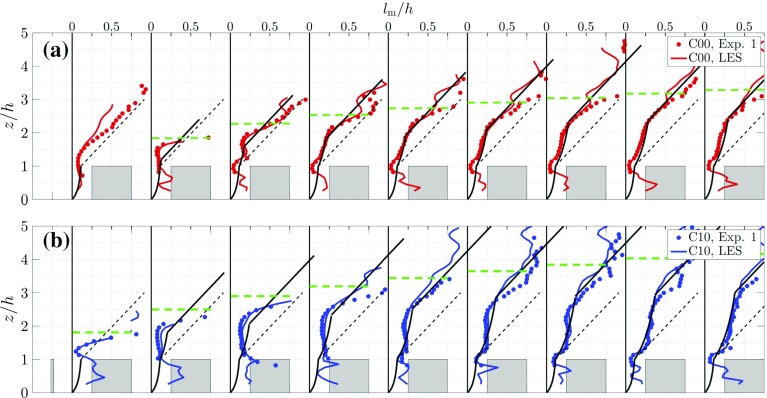
Profiles of the mixing length $l_\mathrm{m}$ in the middle of each street canyon for cases C00 (**a**) and C10 (**b**). The dashed black line is the model of Coceal and Belcher (2004) expressed in Eq. 7, while the modified mixing-length model expressed in Eq. 9 is given by the solid black line. The green dashed line represents the local value for $\delta _i (x)$ determined from the LES data for each case (cf. Figs. 5, 6 and 8)

In Fig. 10 the mixing-length profiles for the C00 and C10 cases, calculated according to Eqs. 5 and 6 , are shown for a distance 0.5*h* in front of the first obstacle row and in the middle of each subsequent street canyon. The gradients of the mean flow in both the experiments and the simulations are calculated using a second-order spatial-regression filter with a filter length of 0.3*h* (Elsinga et al. 2010). Additionally, Fig. 10 shows the models given by Eq. 7 and the updated model given by Eq. 9, which are derived below. Note that the mixing length derived from the measurements in the $y=0$ plane is compared to model results for the spatially-averaged mixing length. When differences between the local and spatially-averaged mixing length exist, this would most likely be in the region where (mean) three-dimensional effects occur, in particular for $z \lesssim h$.

First, the C00 case is discussed. The agreement between the LES and the experimental results is satisfactory within the IBL, i.e. $z<\delta _i$. For the C00 case shown in Fig. 10a it is clear that after a few streets the shape of the mixing-length profile does not change significantly. This result is in line with the observations made by Cheng and Porté-Agel (2015). Inside the canopy ($z<h$), the model of Coceal and Belcher (2004) does not appear to capture the correct trend of $l_\mathrm{m}$, as mentioned before. Figure 10a shows a local peak in the profiles for $l_\mathrm{m}$ at roughly $z/h=0.5$, in accordance with the results of Cheng and Porté-Agel (2015) and Coceal et al. (2006). The profiles of $l_\mathrm{m}$ have a local minimum at roof level. This is the result of the strong shear layer at roof level, resulting in a small mixing length at that location. In the region just above the street canyons, i.e. $1<z/h<1.5$, $l_\mathrm{m}$ increases approximately linearly with height, which is most clearly visible for $x/h\ge 5.5$. This behaviour is indicative of the presence of a logarithmic law in the IBL in which the mixing length scales linearly with $z-d$ (Coceal and Belcher 2004). In the region $1.5<z/h<\delta _i/h$ the mixing length increases approximately linearly, but at a distinctly lower rate compared to the model prediction by Coceal and Belcher (2004). Finally, the mixing length in the outer region of the flow ($z>\delta _i$) again shows a linear increase, indicating the remnants of the logarithmic law region of the original approaching smooth-wall boundary layer. The most important difference between the cases C00 and C10 is found in the region $1.5<z/h<\delta _i/h$. For case C10 (Fig. 10b) $l_\mathrm{m}$ reaches an approximately constant value, and furthermore, the mixing length in this region increases in size when moving downstream.

As a result of the roughness transition a strong shear layer is present above the canopy region that defines the IBL and appears as a well-mixed region, which shows a close resemblance to a plane mixing layer that is characterized by a mixing length that is constant with height and proportional to the mixing-layer depth. It is interesting to note that Belcher et al. (2003) mention the concept of the growth of an internal boundary layer at a roughness transition. However, this concept is not taken into account in the model description. This is likely because relatively sparse canopies are considered for which the strength of the shear layer at the start of the urban canopy does not dominate the flow, and thus the mixing length.

**Fig. 11:**
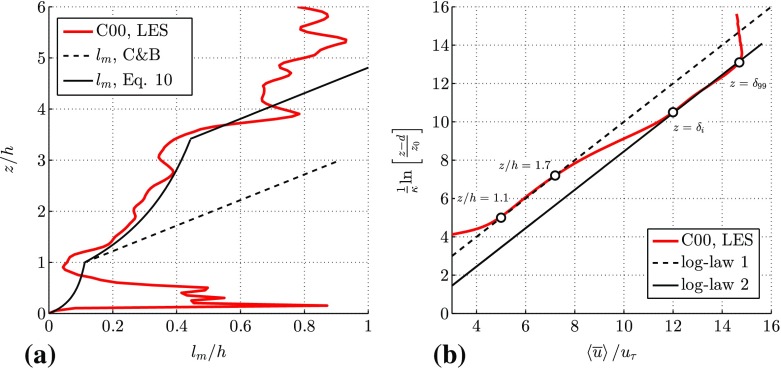
**a** Profile of the mixing length at street 23 in the LES data. The dashed black line depicts the model of Coceal and Belcher (2004) expressed in Eq. 7, while the adjusted mixing-length model according to Eq. 9 is given by the solid black line. **b** The streamwise velocity profile in inner scaling. The dashed line represents the logarithmic law using the local $u_{\tau }$, while the continuous black line is given using the $u_{\tau }$ of the approaching flow

The linear behaviour of $l_\mathrm{m}$, which is indicative of a logarithmic-law behaviour for the mean flow in the regions $1<z/h<1.5$ and $z>\delta _i$, is persistent up to the streamwise extent of the LES domain. Figure 11a shows the mixing length in the 23rd street of the LES domain, while Fig. 11b shows the semi-logarithmic plot of the streamwise velocity profile at the same location. Both C00 and C10 cases indicate the presence of a so-called double logarithmic-layer structure. The bottom logarithmic layer in the range $1<z/h<1.7$ belongs to the IBL over the roughness elements, whereas the upper logarithmic layer ($z/h>4.2$) is the remainder of the approaching-flow boundary layer. These are the regions in Fig. 11a in which $l_\mathrm{m}$ is found to increase linearly with height according to $l_\mathrm{m}=\kappa z$. As the approaching-flow boundary layer has a relatively limited depth of approximately 10*h*, the remainder of the original logarithmic law from the approaching flow boundary layer is only present in the region $3.5<z/h<4$. Above $z/h>4$, $l_\mathrm{m}$ becomes approximately constant, which is the outer layer of the approaching smooth-wall boundary layer in which the scaling $l_\mathrm{m}\propto \kappa (z-d)$ no longer holds.

**Fig. 12:**
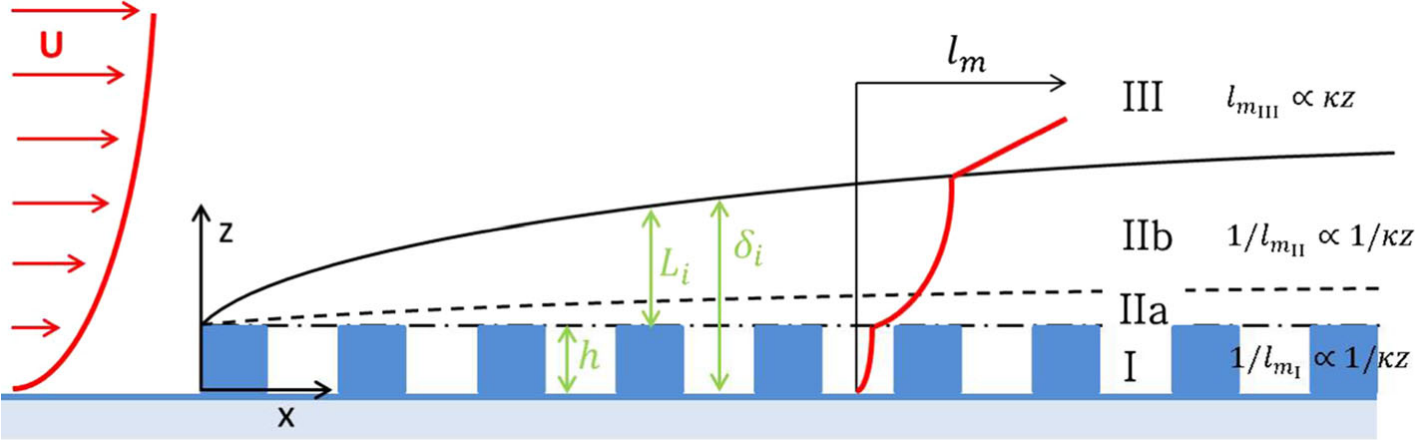
Schematic overview of the different regimes of $l_\mathrm{m}$ in the transition region. The obstacles are shown in blue, the canopy region is denoted by I, the roughness sublayer by IIa, the mixing layer region by IIb and the log-law of the approaching boundary layer by III. The edge of the canopy region is given by the black dash-dotted line, the roughness sublayer is shown by the black dashed line, and the internal boundary layer is given by the solid black line

The mixing-length profiles shown in Figs. 10 and 11a suggest the presence of different regimes that are schematically visualized in Fig. 12. Instead of modelling the mixing length above the urban canopy as $l_\mathrm{m}=\kappa (z-d)$ (Coceal and Belcher 2004), an additional region is present inside the IBL that is governed by a combination of both a linear increase (according to $l_\mathrm{m}=\kappa (z-d)$) just above the canopy and plane mixing-layer-like behaviour in the upper part of the IBL ($l_\mathrm{m}\approx$ constant in *z*, but increasing with *x*). Analogously to the reasoning by Coceal and Belcher (2004) the mixing length in this region (regions IIa and IIb in Fig. 12) can be described by taking the harmonic mean of these two behaviours,

9 $$\begin{aligned} \frac{1}{l_{m_{II}}-\kappa (h-d)} = \frac{1}{\kappa (z-h)} + \frac{1}{l_i}, \end{aligned}$$

where $l_i$ is a length scale that is proportional to the IBL depth, i.e. $l_i=\alpha (\delta _i-h)$. A constant $\alpha =0.2$ appears to give the best match between the measured profiles and the proposed model, which is close to typical values of $\alpha =0.1-0.15$ for mixing layers (Pope 2000). Furthermore, this mixing-length formulation was found to give the best match in the region $1<z/h<0.75\delta _i/h$. The additional term in the denominator on the left-hand side of Eq. 9 originates from the closure relation $l_{m_{\mathrm {II}}}=l_{m_{\mathrm {I}}}$ at $z=h$. As $\delta _i\rightarrow \infty$, the mixing length is dominated by the first term on the right-hand side (r.h.s.) of Eq. 9, and the mixing length returns to $l_\mathrm{m}=\kappa (z-d)$, which is the same form Coceal and Belcher (2004) and others proposed to use above the canopy. Furthermore, for small *z*, $l_\mathrm{m}$ is also dominated by the first term on the r.h.s. of Eq. 9, and $l_\mathrm{m}$ behaves again linearly, which is in accordance with the linear behaviour of $l_\mathrm{m}$ in region IIa. Finally, in region III, that is above the mixing layer, a linear increase of the mixing length with height is applied. From Fig. 10, it is clear that the mixing length according to Eq. 9 provides an accurate description of the mixing length in the region $1<z/h<0.75\delta _i/h$. Especially in the C10 case (see Fig. 10b) the match for the streets further downstream is better compared to the form proposed by Coceal and Belcher (2004). As noted before, the current and previous mixing-length models do not provide accurate results inside dense canopies due to the recirculation regions in the street canyons. However, this is outside the scope of the present investigation. Nonetheless, it is shown that the presence of the IBL should be taken into account to properly model the flow over dense urban canopies.

### Scaling of the Mean Concentration Field

The rural-to-urban roughness transition is characterized by a region where the flow is mainly governed by the strong shear layer emanating from the first row of obstacles or the fence. In the first few streets the flow quickly adapts to the new surface roughness, resulting in a local reduction of the mean streamwise velocity component and a corresponding local vertical velocity component. Coceal and Belcher (2004) proposed a rule of thumb to calculate the length of the adjustment region ($x_0$) as,

10 $$\begin{aligned} x_0=3L_c \mathrm {ln} K, \end{aligned}$$

where $K=(U_h/u_{\tau })/(h/L_c)$, and the length scale $L_c$ is given as,

11 $$\begin{aligned} L_c = \frac{1-\lambda _\mathrm{p}}{\lambda _\mathrm{f}}h. \end{aligned}$$

For the current set-up the adjustment region according to Eq. 10 is approximately 17*h*, i.e. after eight streets the flow at obstacle height has reached a steady value.

**Fig. 13:**
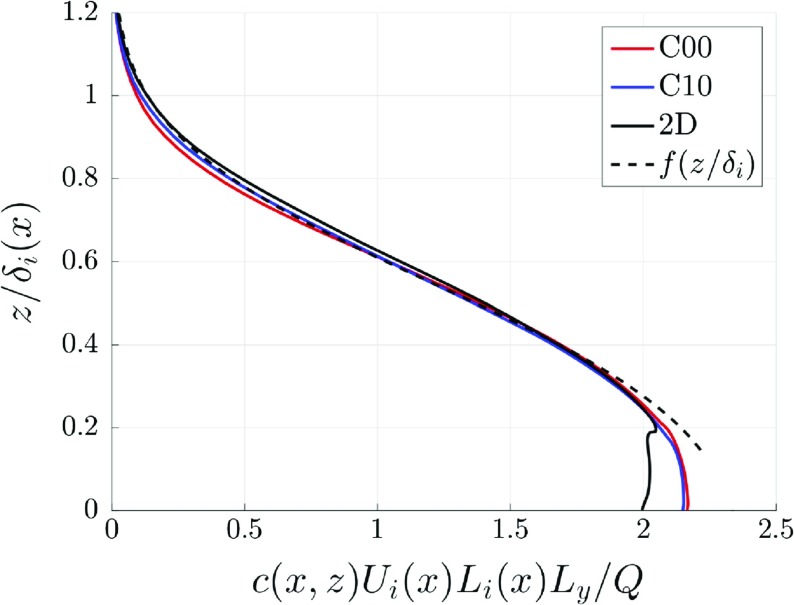
Vertical profiles of the mean concentration from the LES scaled according to Eq. 12 for street 23. The 2D data is taken from Tomas et al. (2017)

The initial pollutant dispersion is governed by the local flow, which is mainly determined by the (local) geometry of the obstacles. However, further downstream the (mean) vertical dispersion is bound by the (mean) shear layer that characterizes the IBL. The vertical extent of the region that bounds the mean concentration field is approximately $L_i=\delta _i -h$ (see Fig. 12). Because $\delta _i$ increases with *x*, $L_i$ also depends on *x*. In addition, the mean advection of pollutants released inside the IBL is governed by the mean streamwise velocity component in the IBL, which can be described by the bulk velocity of the IBL. Here, the streamwise development of the bulk velocity is estimated by the mean streamwise velocity component at half the IBL depth: $U_i(x)\equiv \overline{u}(x,z=\tfrac{1}{2}\delta _i)$. With the length scale $L_i$ and velocity scale $U_i$ the development of the mean concentration field can be described by,

12 $$\begin{aligned} \overline{c}\left( x,z\right) =\frac{Q}{L_y U_i\left( x\right) L_i\left( x\right) }f\left( z/\delta _i\right) , \end{aligned}$$

where $L_y$ is the width of the domain, and *Q* is the constant mass flow rate of the source. Figure 13 shows the vertical profiles of the mean concentration for the C00 and C10 cases, and the 2D case. The vertical coordinate is scaled with the local $\delta _i$, and the concentration is scaled with the local $L_i$ and $U_i$. The results are shown for cases C00, C10, and the 2D case. Although, $U_i$ and $\delta _i$ (and consequently $L_i$) differ for each case, the results collapse onto a single profile using the proposed scaling. A fit for the vertical distribution profile $f\left( z/\delta _i\right)$ is shown by the thick continuous line in Fig. 13. As expected, the profiles start to differ near the surface roughness, where the specific geometry governs the concentration distribution. This scaling means that the spanwise-averaged concentration field can be described by the flow parameters $L_i$ and $U_i$ that depend solely on *x*, while the dependence of *z* is accounted for in the universal function $f\left( z/\delta _i\right)$. In addition, the flow parameters $L_i$ and $U_i$ are most likely related to each other, and they can be found from measurements, simulations, or self-similarity considerations for the flow over a roughness transition.

**Fig. 14:**
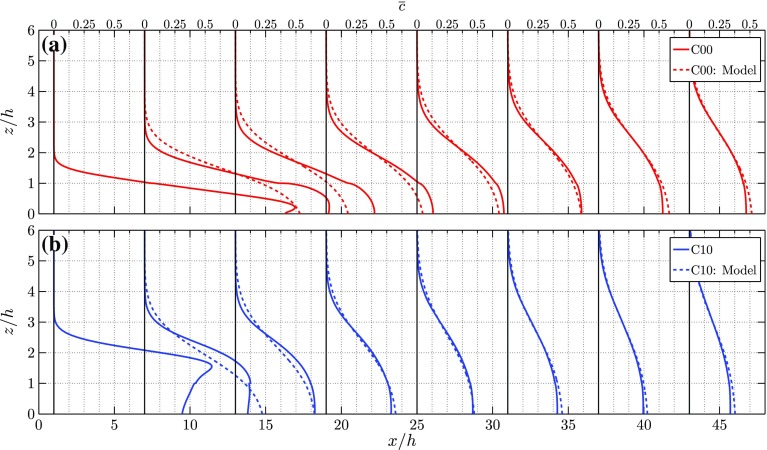
Vertical profiles of the mean concentration for cases C00 and C10 at several downstream locations including model prediction from Eq. 12

Using the fitted profile for $f\left( z/\delta _i\right)$ the prediction of $\overline{c}\left( x,z\right)$ is given in Fig. 14, where for the cases C00 (red lines) and C10 (blue lines) the LES results are shown by the continuous lines, and the model results from Eq. 12 are shown by the thick dashed lines. Close to the start of the urban canopy the model results do not match the LES results. This is expected, since the local velocity field has not yet reached a self-similar state. However, from around street 8 ($x=15h$) onwards the model results agree well the LES results, which indicates that the mean pollutant dispersion is indeed governed by the properties of the IBL.

## Conclusions

Simultaneous PIV and LIF measurements complemented by LES results were employed to study the flow and dispersion behaviour above a rural-to-urban roughness transition. The influence of an additional fence was investigated for various fence heights for the case of a smooth-wall approaching flow at a roughness Reynolds number, $h^+$, of 194 for the simulations and 307 and 213 for the experiments; see Table 2. Both methods predict practically the same velocity and concentration statistics, also in the case where a fence is added. Small differences in the approaching-flow conditions are found to have only a minor influence on the velocity and concentration statistics in the urban canopy region. Secondly, a small non-uniformity of the line source is responsible for the difference in concentration statistics in the first few streets after the roughness transition. Furthermore, the r.m.s. of the canyon concentration fluctuations are considerably higher in the experiments compared to the simulations, as the Schmidt number was also significantly higher in the experiments. Other differences are explained by considering the uncertainty in the data as a result of the finite sampling error of the flow statistics for the given numbers of independent realisations in the experiments and in the LES flow statistics. The differences between the two considered Reynolds number cases are marginal, which suggests that Reynolds number effects are small, as shown previously by Cheng and Castro (2002). A strong shear layer is present at the top of the fence that characterizes the IBL, and with increasing fence height (and thus an increased blockage) a deeper IBL is formed. The disturbance generated by this shear layer determines the downstream development of the IBL, and therefore also the concentration statistics in and above the canopy of the pollutants released upstream of the fence. The concentration statistics show that a higher fence is beneficial to the ventilation in the downstream urban area, as lower pollutant concentration levels are found behind the fence.

The flow in a rural-to-urban transition for dense canopies, i.e. $\lambda _\mathrm{f} \ge 0.25$, can be modelled with a mixing-length model by considering, (1) the roughness geometry, and (2) the IBL development. The effect of the IBL is persistent farther than the streamwise extent of the considered domain for both simulations and experiments. An improved mixing-length model is proposed that is applicable to rural-to-urban transitions for dense urban canopies. Moreover, when $\delta _i$ increases (as $x/h\rightarrow \infty$) this model exhibits similar characteristics to the model of Coceal and Belcher (2004). In addition, the average mean concentration field is found to attain a self-similar profile when scaled with the characteristic velocity and length scale of the IBL, i.e. the IBL bulk velocity and the IBL height. This means that, after an initial adjustment region, the mean concentration field can be described by the characteristics of the IBL solely. Hence, this result emphasizes the conclusion that accurate modelling of the flow and dispersion over rural-to-urban transitions for dense urban canopies requires taking into account the characteristics of the IBL.

Although our investigation is limited to a flow direction normal to the fence and the array of obstacles behind the fence, it provides significant information on the initial development of the IBL and the dispersion of pollutants as a result of the roughness transition. Further studies would be necessary in which also the direction of the flow relative to the fence is varied. The present study validates the LES approach, which can now be extended to investigate other configurations, not only the wind direction with respect to the fence, but also the height of the fence, and distance between the fence and the roughness elements, and the fence and the line source.

## Footnotes

1 Incidentally, this configuration occurs frequently in the Netherlands, where many of the main roadways run north-west–south-east and the dominant wind direction is south-west.
